# Grain Structure Evolution in 6013 Aluminum Alloy during High Heat-Input Friction-Stir Welding

**DOI:** 10.3390/ma16175973

**Published:** 2023-08-31

**Authors:** Alexander Kalinenko, Pavel Dolzhenko, Sergey Malopheyev, Diana Yuzbekova, Ivan Shishov, Vasiliy Mishin, Sergey Mironov, Rustam Kaibyshev

**Affiliations:** 1 Laboratory of Mechanical Properties of Nanoscale Materials and Superalloys, Belgorod National Research University, Pobeda 85, 308015 Belgorod, Russia; kalinenko@bsu.edu.ru (A.K.); dolzhenko_p@bsu.edu.ru (P.D.); malofeev@bsu.edu.ru (S.M.); yuzbekova@bsu.edu.ru (D.Y.); rustam_kaibyshev@bsu.edu.ru (R.K.); 2 Institute of Machinery, Materials, and Transport, Peter the Great St. Petersburg Polytechnic University, 195251 St. Petersburg, Russia; shishov_ia@spbstu.ru (I.S.); mishin_vv@spbstu.ru (V.M.)

**Keywords:** friction-stir welding (FSW), aluminum alloys, microstructure, crystallographic texture, electron backscatter diffraction (EBSD)

## Abstract

This work was undertaken to evaluate the influence of friction-stir welding (FSW) under a high-heat input condition on microstructural evolution. Given the extreme combination of deformation conditions associated with such an FSW regime (including the highest strain, temperature, and strain rate), it was expected to result in an unusual structural response. For this investigation, a commercial 6013 aluminum alloy was used as a program material, and FSW was conducted at a relatively high spindle rate of 1100 rpm and an extremely low feed rate of 13 mm/min; moreover, a Ti-6Al-4V backing plate was employed to reduce heat loss during welding. It was found that the high-heat-input FSW resulted in the formation of a pronounced fine-grained layer at the upper weld surface. This observation was attributed to the stirring action exerted by the shoulder of the FSW tool. Another important issue was the retardation of continuous recrystallization. This interesting phenomenon was explained in terms of a competition between recrystallization and recovery at high temperatures. Specifically, the activation of recovery should reduce dislocation density and thus retard the development of deformation-induced boundaries.

## 1. Introduction

Microstructural control is one of the key issues in friction-stir welding (FSW) [1,2]. This is primarily due to the extremely high sensitivity of the mechanical properties of welded joints to the underlying microstructure. Specifically, the microstructural changes induced by FSW (grain refinement, dissolution of second-phase precipitates, development of crystallographic texture, etc.) may exert a significant influence on weld strength and ductility [1,2].

Moreover, there is also an essential academic interest in FSW-induced microstructures. The unique characteristic of FSW is an exotic combination of very large plastic strains at high temperatures and comparatively high strain rates. As material behavior under such conditions is still not completely clear, investigation of FSWed joints may broaden our understanding of the fundamental microstructural mechanisms.

At present, grain-structure evolution during FSW is most studied in aluminum alloys. The extensive research over the last ~25 years has conclusively demonstrated the complexity of this process in such materials. Specifically, it typically consists of several stages and may involve several different microstructural mechanisms. Those include the geometric effect of strain [3,4,5], continuous recrystallization [6,7,8,9,10,11], or geometric recrystallization [3,12,13,14,15]. Remarkably, the stir zone material may experience secondary deformation associated with the tool shoulder [16]. This effect is most pronounced at the upper weld surface and may produce a fine-grained microstructural layer in this area [17]. After FSW, the welded material often experiences a static microstructural coarsening upon cooling from FSW temperature to ambient conditions [3,18,19,20].

It is well accepted that microstructural changes during FSW are significantly influenced by the thermal conditions of welding. However, the underlying mechanism is not completely clear. In fact, it is only well known that an increase in the FSW heat input leads to grain coarsening within the stir zone [1].

In this context, of particular interest is an examination of FSW under high heat input conditions. Such FSW is usually realized using a combination of a high spindle rate and a low feed rate, thus being characterized by the highest welding temperature, the largest plastic strain [16], and, perhaps, the highest strain rate. In other words, the high-heat-input FSW comprises the most extreme combination of deformation conditions.

To the best of the authors’ knowledge, this FSW range has not been studied systematically so far. Thus, to broaden our fundamental understanding of microstructural aspects of FSW, the present study was focused on the elucidation of a specific character of grain-structure evolution at high heat input conditions.

## 2. Materials and Methods

The commercial aluminum alloy 6013 was selected as a program material for the present study (Table 1). This alloy belongs to the 6xxx series of aluminum alloys, whose FSW behavior is studied relatively well. Despite being a particle-containing aluminum alloy, this material is suitable for the purpose of the present study (i.e., examination of grain-structure evolution during FSW) because the second-phase particles are expected to completely dissolve at the high–heat input condition.

The program material was produced by semi-continuous casting using a KREMIA laboratory casting machine. The cast ingot was then homogenized at 550 °C for 4 h, with subsequent cooling in air. To enhance the effect of the high–heat input FSW on microstructure evolution, the program material was subjected to extensive cold rolling prior to the welding. To this end, the homogenized ingot was sliced along its longitudinal axis and then rolled at ambient temperature to a total thickness reduction of ≈80%. The produced material had a typical work-hardened microstructure, which consisted of pancake-shaped grains, a dense subboundary structure, and a high dislocation density (Supplementary Figure S1a). Crystallographic texture was dominated by the typical rolling components, including S {123}<634>, Copper {112}<111>, Brass {110}<112>, and Cube {001}<100> orientations (Supplementary Figure S1b). Throughout the manuscript, this material condition was referred to as *base material.*

The 2-mm-thick sheets of the base material were friction-stir welded in a *bead-on-plate* configuration using a commercial AccuStir FSW machine. To provide a high–heat input condition, a spindle rate of 1100 rpm and a feed rate of 0.5 inches per minute (≈13 mm/min) were employed. Given the relatively wide FSW processing window for aluminum alloys, such an extremely low feed rate is very rarely used in welding practice, to the best of the author’s knowledge. Moreover, to reduce heat loss during FSW, a 2-mm-thick Ti-6Al-4V interlayer was used between the aluminum plate and the steel backing plate. 

Per analogy with the previous work [21], the welding tool was manufactured from tool steel and consisted of a concave-shaped shoulder of 12.5 mm in diameter and an M5-threaded probe of 1.9 mm in length. FSW was conducted under the plunge-depth control mode, while the distance between the probe tip and the interlayer material was maintained to be as small as ≈50 μm. A typical convention of FSW geometry was adopted, which included welding direction (WD), normal direction (ND), and transverse direction (TD). 

**Figure 1:**
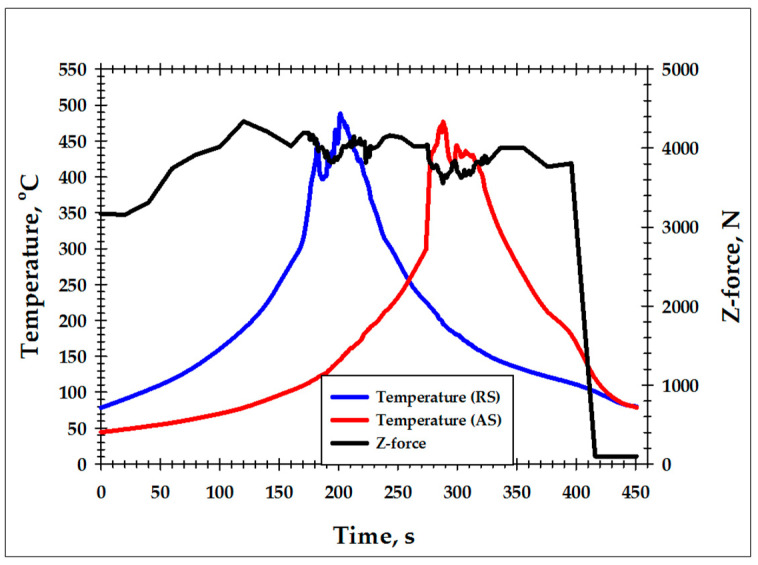
The FSW thermal cycles recorded at the retreating side (RS) and the advancing side (AS) and the evolution of Z-force during FSW.

To record the weld thermal cycle, two thermocouples were placed beneath the aluminum plane in close proximity to the probe tip. A schematic of the thermocouple layout is given in supplementary Figure S2. The measured peak temperature was close to ~500 °C or ≈0.9 T_m_ (where T_m_ is the melting point) (Figure 1); hence, the actual temperature within the stir zone was likely even higher. Moreover, the cooling rate was also found to be low; specifically, it took about 3.5 min to cool the weld from ≈500 to ≈100 °C (Figure 1). Thus, despite the fact that the term “high heat input” is not strictly defined in FSW, it could be concluded that this welding condition was provided in the present study.

On the other hand, the tracking of the Z-force evolution showed that material flow during FSW was relatively unstable. In addition to the expected fluctuations near the thermocouple positions, the loading force was found to significantly vary along the weld path (Figure 1).

For microstructural examinations, the produced weld was sectioned in the ND × TD plane. Attempting to provide a link with temperature measurements and avoid the influence of the unstable material flow, the microstructural sample was machined between the thermocouple positions (Figure 1). It was then mechanically polished in a conventional fashion, with the final step comprising 24-h vibratory polishing using the commercial OP-S suspension.

Microstructural observations were performed using the electron backscatter diffraction (EBSD) technique. To this end, an FEI Quanta field-emission-gun scanning electron microscope (FEG-SEM) (FEI Company, OR, US) was employed. The FEG-SEM was equipped with TSL OIM^TM^ software (Version 8.0) and operated at an accelerating voltage of 20 kV. To provide a broad view of the FSW-induced microstructure, a series of sample-scale maps with a comparatively coarse scan step size of 2 μm were acquired from the entire stir zone. On the other hand, to get a close inspection of microstructures in particular areas of interest, a number of higher-resolution maps with scan step sizes of 0.5 and 0.2 μm were also obtained. To improve the fidelity of EBSD data, the small grains comprising either one or two pixels were “cleaned” from the maps using the standard grain-dilation option of EBSD software (Version 8.0). Considering the limited angular accuracy of EBSD, grain boundaries with a misorientation angle below 2° were excluded from consideration. To differentiate the low- and high-angle boundaries (LABs and HABs, respectively), a 15-degree threshold was applied. Grain size was measured using the equivalent circle-diameter approach [22].

To evaluate the effect of the post-weld aging treatment, some of the welded joints were artificially aged at 190 °C for 4 h [23].

In order to get a broad insight into the microstructure distribution within the welded material, a series of microhardness profiles were measured across the weld’s mid-thickness. The Vickers microhardness data were collected employing a Wolpert 402MVD microhardness tester by applying a load of 200 g, a dwell time of 10 s, and a step size of 0.5 mm. 

## 3. Results

### 3.1. Microhardness Distribution

**Figure 2:**
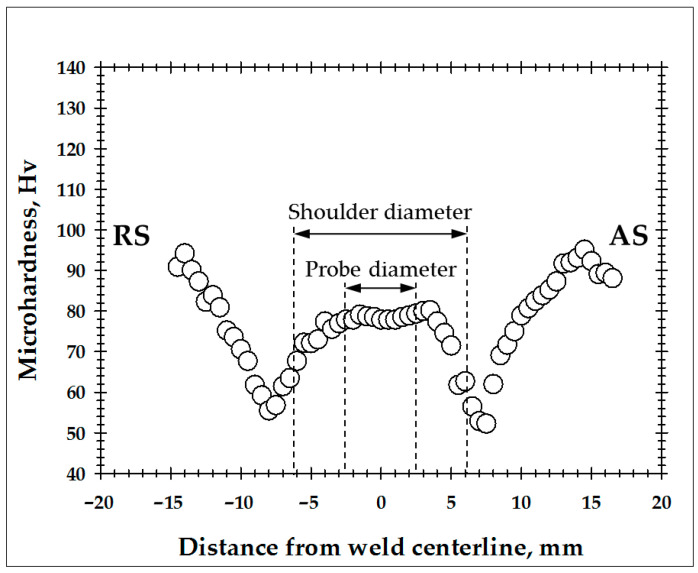
Microhardness profile measured across the midthickness of the weld cross-section. RS and AS are retreating side and advancing side, respectively.

In order to identify the microstructural zones produced during FSW, a microhardness profile was measured across the welded joint, as shown in Figure 2. For clarity, the dimensions of the tool probe and the tool shoulder were also indicated. In a first approximation, those point to the heat-affected zone and the stir zone, respectively.

Near the edge of the tool shoulder (i.e., virtually in the heat-affected zone), a pronounced material softening was evident. Specifically, the hardness decreased approximately by half, from ≈90 to ≈50 Hv. Remarkably, the softening effect was most prominent on the advancing side of the weld. By approaching the tool probe, the hardness tended to increase back and then saturated at ≈80 Hv within the stir zone. 

The revealed variations in microhardness implied significant changes in the underlying microstructure. To ascertain those, EBSD maps were acquired from the heat-affected zone and the thermo-mechanically affected zone and then considered in the following two sections.

### 3.2. Heat-Affected Zone

**Figure 3:**
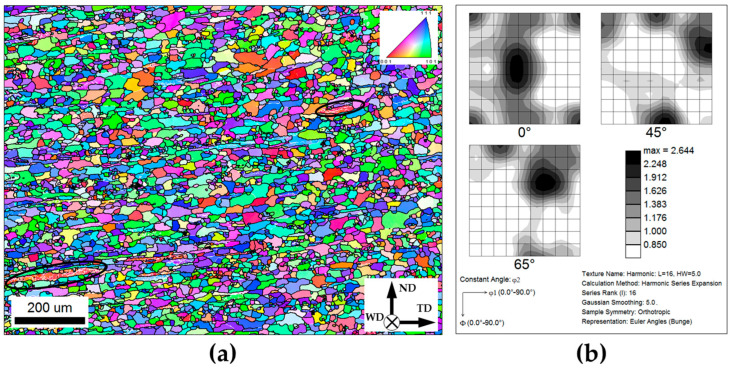
Microstructure and crystallographic texture of the *heat-affected zone*: (**a**) EBSD orientation map and (**b**) φ_2_ = 0°, φ_2_ = 45°, and φ_2_ = 65° sections of the orientation distribution function. In (**a**), grains are colored according to their crystallographic orientations relative to the welding direction (the orientation color code is shown in the top right corner of (**a**)), while LABs and HABs are depicted as white and black lines, respectively; WD, ND, and TD are the welding direction, normal direction, and transverse direction, respectively. Note: The selected areas in (**a**) exemplify the retained remnants of the initial cold-rolled microstructure.

The EBSD orientation map taken approximately beneath the shoulder edge is shown in Figure 3a. The individual grains in the map are colored according to their crystallographic orientations relative to the welding direction (the color code triangle is shown in the top right corner), while LABs and HABs are depicted as white and black lines, respectively.

It is seen that the initial heavily rolled microstructure was almost completely replaced by relatively coarse (~100 μm) low-aspect-ratio grains, which typically contained no LAB substructure. Only a few fragments of the initial cold-rolled grains had survived within the microstructure (several examples were circled). These observations undoubtedly indicated that the material in the heat-affected zone had experienced recrystallization. 

In order to get additional insight into the recrystallization process, the evolved crystallographic texture was also analyzed. To this end, orientation data were derived from the EBSD map and arranged as an orientation distribution function (ODF). The characteristic sections of the ODF are shown in Figure 3b, while the volume fractions of the dominant texture components are summarized in Table 2. It is seen that the static recrystallization retained the initial rolling texture but reduced its intensity.

The recrystallization revealed within the heat-affected zone explains well the drastic material softening in this area (Figure 2). Given the relatively high isotropy inherent to aluminum alloys, it is highly likely that the softening effect was primarily associated with the elimination of dislocation substructure rather than with texture changes.

### 3.3. Thermo-Mechanically Affected Zone

**Figure 4:**
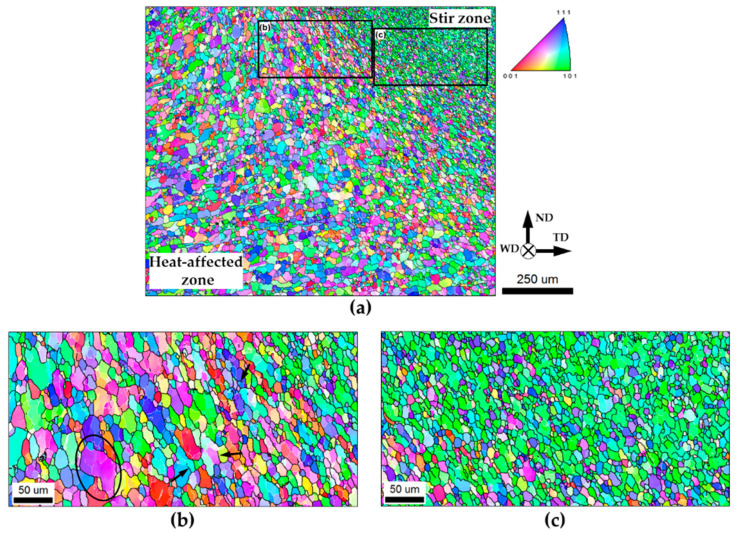
(**a**) EBSD orientation map showing microstructural evolution in the *thermo-mechanically* affected zone, with selected areas given at higher magnifications in (**b**,**c**). Individual grains in the map are colored according to their crystallographic orientations relative to the welding direction (the orientation color code is shown in the top right corner of (**a**)), while LABs and HABs are depicted as white and black lines, respectively. WD, ND, and TD are the welding direction, normal direction, and transverse direction, respectively.

The EBSD orientation map taken from a transition region between the heat-affected zone and the stir zone is shown in Figure 4a. The selected areas of the map are given at higher magnifications in Figure 4b,c. Again, individual grains in all cases were colored according to their crystallographic orientations relative to the welding direction, and LABs and HABs are depicted as white and black lines, respectively.

Near the inner edge of the heat-affected zone, recrystallized grains were geometrically reoriented/sheared, and drawn in a common direction, which lay approximately parallel to the edge of the stir zone (Figure 4a). This effect is often observed in friction-stirred materials [3,5] and is usually attributed to the geometric effect of strain imposed by the rotating tool. Within the sheared grains, a subgrain structure had developed (selected area in Figure 4b). The local subboundary segments often exhibited misorientation angles exceeding 15° (several examples are arrowed in Figure 4b), thus evidencing a gradual transformation of LABs to HABs. The progressive development of this process eventually broke up the recrystallized grains into a fine-grained structure that contained a large proportion of LABs (Figure 4c). Remarkably, the evolution of the deformation-induced boundaries was accompanied by significant crystallographic rotations, seen as variations in orientation contrast in Figure 4b. This process ultimately resulted in the development of the green-colored grains within the stir zone (Figure 4a,c), which implied the formation of a specific crystallographic texture. In greater detail, this issue is considered in Section 3.5.

Thus, the microstructural process within the thermo-mechanically affected zone fits the definition of *continuous recrystallization*. This result was consistent with a number of reports in the FSW literature [6,7,8,9,10,11]. On the other hand, the concomitant grain refinement was in agreement with the substantial material hardening revealed in this microstructural region.

### 3.4. Microstructure Distribution within the Stir Zone

**Figure 5:**
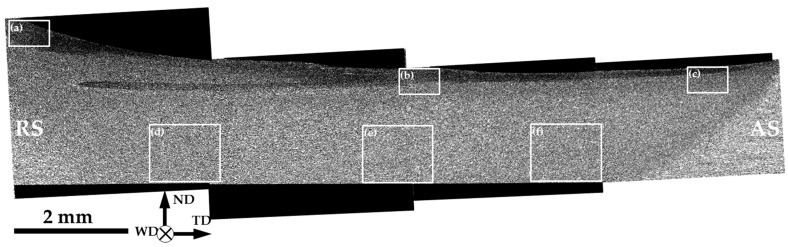
The sample-scale EBSD grain boundary map shows microstructure distribution within the stir zone. For simplicity, only HABs are shown. AS, RS, ND, WD, and TD are the advancing side, retreating side, normal direction, welding direction, and transverse direction, respectively. Note: The labeled areas are shown at higher magnifications in Figure 6.

To evaluate the broad aspects of microstructure distribution within the stir zone, a series of sample-scale EBSD maps were obtained. Those were then arranged in a panoramic grain-boundary map, as shown in Figure 5. In this map, HABs are depicted as black lines, while LABs are omitted for simplicity.

An inhomogeneous microstructure distribution is evident. In a first approximation, two macro-scale subareas could be defined within the stir zone, viz. (i) a relatively fine-grained surface layer and (ii) a coarse-grained remaining part. The formation of a fine-grained surface layer in the stir zone is sometimes reported in FSW literature and is typically attributable to the stirring action of the tool shoulder [17]. 

**Figure 6:**
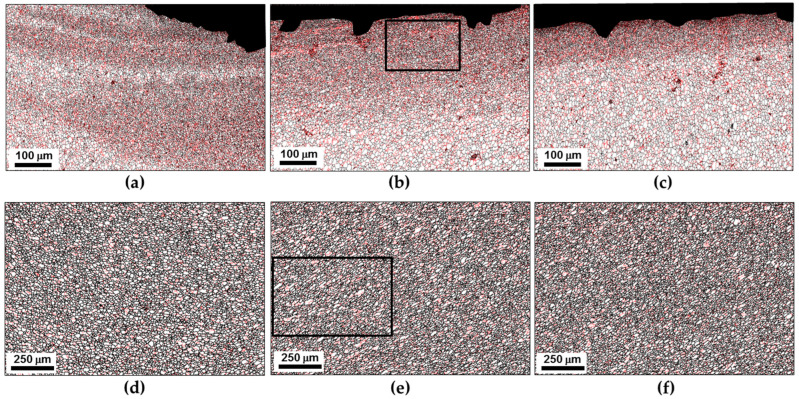
The selected portions of the EBSD grain-boundary map in Figure 5 show microstructures in different locations within the stir zone: (**a**) upper surface, advancing side, (**b**) upper surface, center, (**c**) upper surface, retreating side, (**d**) weld nugget, retreating side, (**e**) weld nugget, center, and (**f**) weld nugget, retreating side. Note differences in scales. In all cases, LABs and HABs are depicted as red and black lines, respectively. Note: The selected areas in (**b**,**e**) are given at higher magnifications in Figure 8.

**Figure 7:**
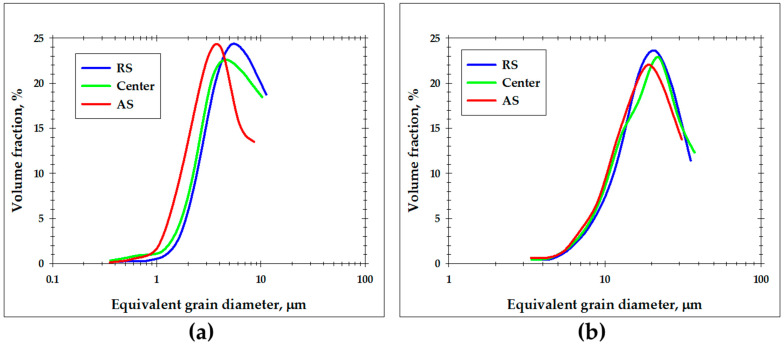
Grain-size distributions measured in different locations of the stir zone: (**a**) upper surface and (**b**) weld nugget. Note: The grain size data were derived from EBSD maps shown in Figure 6.

In order to get a closer inspection of the evolved microstructures, several local areas of the sample-scale map in Figure 5 were given at higher magnifications in Figure 6. The grain-size statistics derived from these areas are summarized in Figure 7. It is seen that the fine-grained surface layer was most pronounced at the retreating side of the stir zone (Figure 6a) but tended to narrow towards the advancing side (Figure 6b,c). Moreover, a subtle decrease in the average grain size was also found (Figure 7a). In contrast, the grain size in the nugget zone was relatively stable (Figure 7b), and no significant changes in microstructure morphology were found (Figure 6d–f).

**Figure 8:**
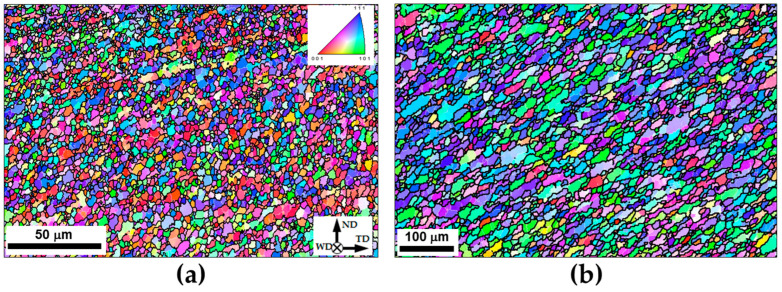
High-resolution EBSD orientation maps showing typical microstructure evolved (**a**) at the upper surface of the stir zone and (**b**) within the weld nugget. In both maps, individual grains are colored according to their crystallographic orientation relative to the welding direction (the color code triangle is shown in the top right corner of (**a**)); LABs and HABs are depicted as white and black lines, respectively. The reference frame for both maps is shown in the bottom right corner of (**a**); ND, WD, and TD are the normal direction, welding direction, and transverse direction, respectively. Note the difference in scales.

To provide additional insight into the microstructure, high-resolution EBSD orientation maps were taken from the surface layer and the nugget zone and shown in Figure 8. In both cases, an important characteristic of the microstructures was a significant fraction of the retained coarse grains with developed subgrain structure. This observation implied the incompleteness of the recrystallization process within the stir zone. Considering the high–heat input condition of FSW employed in the present study (which involved the highest temperature and perhaps a comparatively large plastic strain), this result appears to be non-trivial.

### 3.5. The Distribution of Crystallographic Texture within the Stir Zone

**Figure 9:**
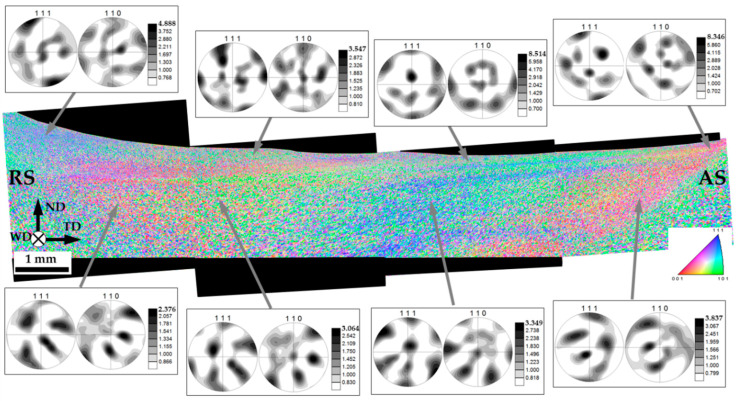
The sample-scale EBSD orientation maps with a series of the measured 111 and 110 pole figures, which show crystallographic texture in different locations within the stir zone. In the map, individual grains are colored according to their crystallographic orientation relative to the WD (the color code triangle is shown in the bottom right corner). RS, AS, ND, WD, and TD are retreating side, advancing side, normal direction, welding direction, and transverse direction, respectively.

To examine the distribution of crystallographic texture within the stir zone, the acquired EBSD data were rearranged as a sample-scale EBSD orientation map (Figure 9). 

It is well accepted that FSW results in crystallographic textures that are close to the simple-shear ones [24]. In face-centered cubic metals (including aluminum alloys), those are usually represented using 111 and 110 pole figures, where the 111 pole figure exhibits an orientation of a shear plane normal, while the 110 pole figure indicates an orientation of shear direction [24]. Accordingly, orientation data were derived from several areas of the surface layer and the nugget zone, arranged as 111 and 110 pole figures and also shown in Figure 9.

It is seen that traversing from the retreating side of the stir zone to the advancing side leads to a rotation of the pole figures around their vertical axis (i.e., virtually around the normal direction). This effect is well-known in FSW literature [24], and it is usually associated with a rotation of the shear direction within the stir zone. It is commonly believed that the shear direction is tangential to the surface of the rotating tool, and thus it follows a semi-circular line within the stir zone. Specifically, it should be nearly opposite to the welding direction at the retreating side, be along with the transverse direction at the central part of the stir zone, and be collinear with the welding direction at the advancing side. The orientation of the shear plane is less evident in a general case. Nevertheless, it is often believed to be close to the plane of the tool shoulder in the upper section of the stir zone and nearly parallel to the cylindrical surface of the tool probe in the weld nugget. As a result, an appropriate texture analysis requires a proper rotation of the measured pole figures. In the weld section examined in the present work (i.e., the ND × TD plane), the experimental pole figures should be rotated around the normal direction to align their horizontal axes with the shear direction. Then, those should be titled around the transverse direction to coincide their vertical axes with the shear plane normal. 

**Figure 10:**
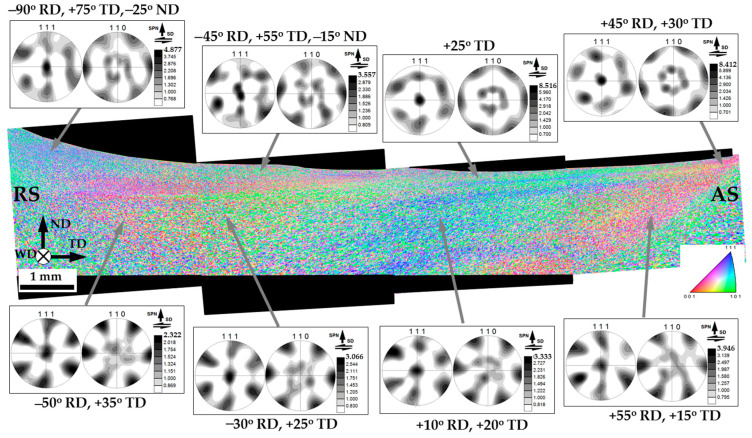
The sample-scale EBSD orientation maps with a series of the rotated 111 and 110 pole figures, which show crystallographic texture in different locations within the stir zone. In the map, individual grains are colored according to their crystallographic orientation relative to the WD (the color code triangle is shown in the bottom right corner). RS, AS, ND, WD, and TD are retreating side, advancing side, normal direction, welding direction, and transverse direction, respectively. Note: The pole figures were rotated in order to align their reference frames with the local geometry of the simple-shear deformation; the applied rotations are indicated at the pole figures. SPN and SD are the shear plane normal and the shear direction, respectively.

Hence, the measured pole figures were appropriately rotated to align their geometry with the presumed geometry of local shear strain. The rotated pole figures are shown in Figure 10. The applied rotations were indicated beneath the pole figures.

Of particular interest was the observation that the rotation around the transverse direction was typically small. In most cases, it did not exceed 35° (Figure 10). This result implied that the shear plane within the entire stir zone (including the weld nugget) was closer to the surface of the tool shoulder than to the surface of the tool probe. In other words, material flow within the entire stir zone was mainly governed by the tool shoulder rather than the tool probe. This interesting phenomenon is discussed in Section 4. 

In all cases, the textural patterns revealed in the rotated pole figures could be described in terms of $B/\bar{B}\{112\}<110>$ simple shear texture; in some cases, they were additionally complemented by {hkl}<110>-fiber (Figure 10). Such textures are typically reported to form in FSWed aluminum alloys [24]. 

Remarkably, the material in the upper section of the stir zone typically exhibited a stronger but more complex textural pattern than that in the weld nugget (Figure 10). This observation was consistent with the formation of the fine-grained layer (Figure 5), thus reflecting the complex character of material flow in this area [16]. 

Another interesting point was the revealed tendency for the texture to strengthen from the retreating side towards the advancing side (Figure 10). The origin of this phenomenon was not clear, however. 

## 4. Discussion

### 4.1. Microstructural Evolution during FSW

The purpose of this work was the examination of the effect of the high–heat input FSW on microstructural evolution and material flow. From the experimental results summarized in Section 3, the following four important findings were derived:

(i) The base material underwent almost complete static recrystallization in the heat-affected zone. Thus, the initial microstructure was fundamentally altered *before* its direct contact with the FSW tool.
(ii) Material flow during FSW was primarily governed by the tool shoulder.
(iii) FSW resulted in the formation of a pronounced layer of fine-grained microstructure at the upper surface of the stir zone.
(iv) The microstructure within the stir zone contained a significant fraction of the survived remnants of coarse grains, i.e., the recrystallization process was far from being completed.

Hereafter, each of these effects will be considered in detail. 

The static recrystallization in the heat-affected zone was obviously related to the high-temperature condition of the applied FSW process. On the other hand, this effect was also significantly contributed to by the heavily deformed state of the base material. It is expected that a material in a well-annealed initial temper would not experience recrystallization (or even notable grain growth) during FSW in the same thermal environment. Therefore, the static recrystallization in the heat-affected zone was unlikely an intrinsic characteristic of the high–heat input FSW. 

The dominant role of the tool shoulder in material flow during FSW was presumably associated with a comparatively large plunging depth of the shoulder into the welded material. This increased the friction effect between the shoulder and the material and thus promoted both an increase in the welding temperature and the contribution of the shoulder to material flow. However, the scale of the latter phenomenon was also substantially influenced by the relatively small thickness of the welded workpieces employed in the present study (i.e., 2 mm). It is fairly likely that an increase in material thickness (and the concomitant increment in the length of the tool probe) will reduce the contribution of the tool shoulder to global material flow. Hence, the shoulder-induced material flow was probably also not inherent to the high–heat input FSW. 

The development of the pronounced fine-grained layer at the upper surface of the stir zone was obviously attributable to the stirring action of the tool shoulder. As shown in some experimental works [17] and numerical simulations [16], the stir zone material should experience a secondary strain induced by the tool shoulder; this effect was due to the backward tilting of the FSW tool. This event is characterized by an extreme combination of large plastic strain, the highest temperature, and (presumably) the highest strain rate, and thus should lead to a drastic microstructural modification at the upper weld surface. Under a high–heat input condition, the stir zone material should be comparatively soft. Hence, the secondary deformation should result in a pronounced surface layer, as has indeed been found in the present study (Figure 5). This surface layer may provide abnormal grain growth during post-weld annealing treatment [17], thus playing an important role in the service performance of welded joints. 

The continuous recrystallization within the stir zone was in good accordance with the scientific literature [6,7,8,9,10,11]. On the other hand, the revealed incompleteness of this process was perhaps the most interesting result. Given the high–heat input condition of FSW, which implies the largest strain at the highest temperature, this effect appears to be surprising. One of its possible explanations may be an enhancement of recovery at high temperatures. This should reduce dislocation density and thus inhibit the development of deformation-induced boundaries. In other words, the observed retardation of recrystallization within the stir zone was likely a result of competition between the recrystallization and the recovery. As this phenomenon is most pronounced at the highest temperatures, it should be intrinsic to the high–heat input FSW.

### 4.2. The Post-Weld Aging Behavior

As the 6013 aluminum alloy belongs to heat-treatable materials, its service properties rely heavily on aging treatment. Hence, to estimate the industrial performance of the high-heat-input FSW weld, its aging behavior was evaluated. The obtained results are outlined in the present section.

**Figure 11:**
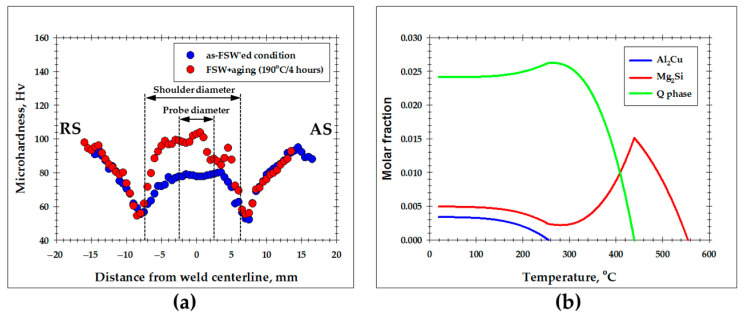
(**a**) The effect of post-weld aging treatment on the microhardness profile measured across the midthickness of the weld cross-section; (**b**) Thermo-Calc predictions of the temperature dependence of the secondary phase fractions in 6013 aluminum alloy. In (**a**), RS and AS denote retreating and advancing sides, respectively.

The microhardness profile measured across the midthickness of the aged weld is shown in Figure 11a. From the comparison with the profile measured in the as-FSW’ed condition, it was found that the aging treatment exerted no significant influence on the material *outside* the diameter of the tool shoulder. On the other hand, significant material strengthening was revealed *within* the shoulder diameter, with the effect being most pronounced within the stir zone.

It is expected from the above observations that the tensile behavior of the aged FSW joint should be essentially degraded by material softening within the heat-affected zone (Figure 11a). Thus, an elucidation of the negligible aging effect in this microstructural area is of interest. To assist in the interpretation of the underlying microstructural changes, the evolution of the constituent secondary particles was predicted for 6013 aluminum alloy using ThermoCalc calculations with the TCAl8:Al-Alloys v.8.1 database. The simulation results are summarized in Figure 11b.

Given the long-time and high-temperature character of the weld thermal cycle (Figure 1), it is possible that the particles could *precipitate* in the heat-affected zone during FSW. As discussed in Section 2, the peak FSW temperature within the stir zone presumably exceeded 500 °C. Assuming that the temperature in the heat-affected zone ranged between 200 and 450 °C, it could be suggested that the secondary particles in this region were represented by a mixture of Al_2_Cu, Mg_2_Si, and Q phases (Figure 11b). Accordingly, the post-weld aging at 190 °C provided only a small precipitation effect, and therefore material strength remained nearly unchanged (Figure 11a).

On the other hand, considering the highest FSW temperature within the stir zone, the precipitated particles within this region may then *dissolve*, leaving only a minor fraction of the Mg_2_Si phase (Figure 11b). Hence, the post-weld aging at 190 °C should promote extensive precipitation of Al_2_Cu and Q phases (Figure 11b), thus giving rise to the revealed hardening effect (Figure 11a). 

Despite the fact that the above hypotheses seem reasonable, it is important to emphasize that they are based only on ThermoCalc predictions and thus lack experimental verification. Hence, appropriate microstructural observations of the particle behavior are necessary. However, this issue is outside the scope of the present work. 

## 5. Summary

This work was undertaken to evaluate the influence of the high–heat input condition of FSW on microstructural evolution and material flow. Considering the extreme combination of deformation conditions inherent to such a welding regime (including an extremely large plastic strain, the highest temperature, and a high strain rate), it was expected to reveal an unusual structural response. The most important findings included: (i) the formation of a pronounced fine-grained layer at the upper weld surface; and (ii) a retardation of the recrystallization process within the stir zone. 

The development of the surface layer was attributed to the material softening within the stir zone at elevated temperatures. As a result, the secondary strain induced by the tool shoulder has to result in essential microstructural modifications at the upper weld surface. 

The retardation of continuous recrystallization within the stir zone was explained in terms of the concurrent influence of recovery. The activation of the latter mechanism at elevated temperatures should reduce dislocation density and thus slow down the development of deformation-induced boundaries.

## Figures and Tables

**Table 1:** The nominal chemical composition of 6013 aluminum alloy (wt.%).

Al	Mg	Si	Cu	Mn	Fe	Zn	Cr	Ti
Bal.	0.8–1.2	0.6–1.0	0.6–1.1	0.2–0.8	≤0.5	≤0.25	≤0.1	≤0.1

**Table 2:** Effect of static recrystallization in the heat-affected zone on the volume fraction of the dominant texture components.

Texture Component	Volume Fraction, %	
	Base Material	Heat-Affected Zone
Copper {112}<111>	7.7	6.7
S {123}<634>	20.3	13.2
Brass {011}<211>	9.4	7.5
Cube {001}<100>	3.3	3.2

## Data Availability

Data will be made available on request.

## Supplementary Materials

The following supporting information can be downloaded at: https://www.mdpi.com/article/10.3390/ma16175973/s1, Figure S1. Microstructure and crystallographic texture of base material: (a) selected portion of EBSD grain-boundary map and (b) φ_2_ = 0°, φ_2_ = 45°, and φ_2_ = 65° sections of orientation distribution function. In (a), low-angle boundaries and high-angle boundaries are depicted as red and black lines, respectively; RD, ND and TD are rolling direction, normal direction, and transverse direction, respectively. Figure S2. Schematic showing the thermocouple layout. Scale: mm.
