# Supplementary material for: Grain Structure Evolution in 6013 Aluminum Alloy during High Heat-Input Friction-Stir Welding
Source: Materials (Basel). 2023 Aug 31;16(17):5973. doi: 10.3390/ma16175973 (PMC10488852; doi:10.3390/ma16175973)
Supplement: Supplementary file 1 [file materials-16-05973-s001.zip › materials-2544013-supplementary.pdf]

materials

Supplementary Material

Grain Structure Evolution in 6013 Aluminum Alloy during High Heat-Input Friction-Stir Welding

Alexander Kalinenko [1], Pavel Dolzhenko [1], Sergey Malopheyev [1], Diana Yuzbekova [1], Ivan Shishov [2], Vasiliy Mishin [2], Sergey Mironov [1,*] and Rustam Kaibyshev [1]

1 Laboratory of Mechanical Properties of Nanoscale Materials and Superalloys, Belgorod National Research University, Pobeda 85, 308015 Belgorod, Russia; kalinenko@bsu.edu.ru (A.K.); dolzhenko_p@bsu.edu.ru (P.D.); malofeev@bsu.edu.ru (S.M.); yuzbekova@bsu.edu.ru (D.Y.); rustam_kaibyshev@bsu.edu.ru (R.K.)

2 Institute of Machinery, Materials, and Transport, Peter the Great St. Petersburg Polytechnic University, 195251 St. Petersburg, Russia; shishov_ia@spbstu.ru (I.S.); mishin_vv@spbstu.ru (V.M.)

* Correspondence: mironov@bsu.edu.ru; Tel.: +7-4722-585456

Citation: Kalinenko, A.; Dolzhenko, P.; Malopheyev, S.; Yuzbekova, D.; Shishov, I.; Mishin, V.; Mironov, S.; Kaibyshev, R. Grain Structure Evolution in 6013 Aluminum Alloy during High Heat-Input Friction-Stir Welding. *Materials* **2023**, *16*, 5973. https://doi.org/10.3390/ma16175973

Academic Editors: Małgorzata Karolus and Sabina Lesz

Received: 21 July 2023
Revised: 22 August 2023
Accepted: 25 August 2023
Published: 31 August 2023

Copyright: © 2023 by the authors. Licensee MDPI, Basel, Switzerland. This article is an open access article distributed under the terms and conditions of the Creative Commons Attribution (CC BY) license (https://creativecommons.org/licenses/by/4.0/).

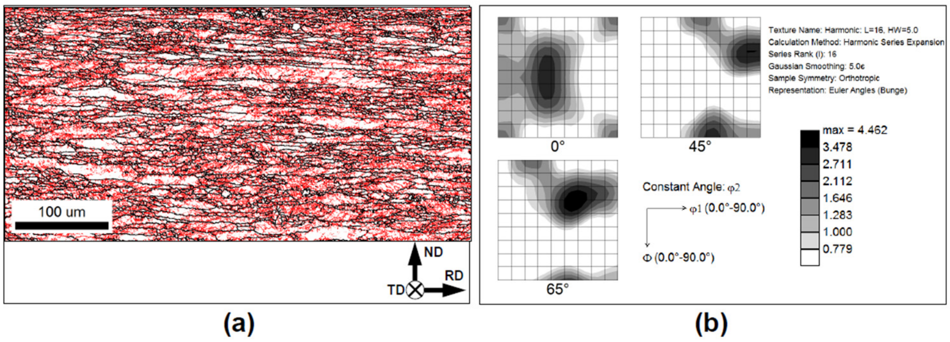

Figure S1. Microstructure and crystallographic texture of base material: (a) selected portion of EBSD grain-boundary map and (b) j2=0o, j2=45o, and j2=65o sections of orientation distribution function. In (a), low-angle boundaries and high-angle boundaries are depicted as red and black lines, respectively; RD, ND and TD are rolling direction, normal direction, and transverse direction, respectively.

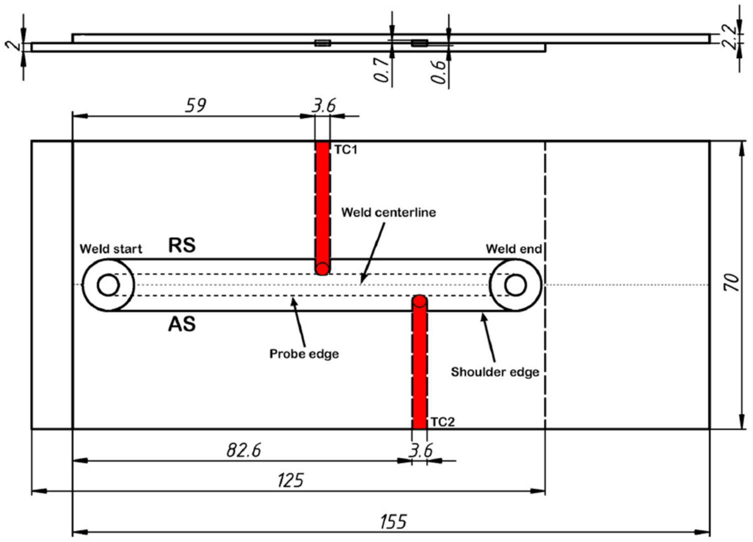

Figure S2. Schematic showing the thermocouple layout. Scale: mm
